# Changes in the Microbiome of Sugarcane (*Saccharum* spp. Hybrids.) Rhizosphere in Response to Manganese Toxicity

**DOI:** 10.3390/life13101956

**Published:** 2023-09-25

**Authors:** Qiuyue Li, Qiuliang Cai, Linjuan Pan, Xinlian Tang, Guizhi Ling, Yanyan Wei, Xiaofeng Li, Shu Yang

**Affiliations:** 1 State Key Laboratory for Conservation and Utilization of Subtropical Agro-Bioresources, College of Agriculture, Guangxi University, Nanning 530004, Chinatxl@gxu.edu.cn (X.T.);; 2 Agriculture and Food Engineering College, Baise University, Baise 533000, China; 3 Institute for New Rural Development, Guangxi University, Nanning 530004, China

**Keywords:** rhizosphere microbes, abiotic stress, manganese toxicity, sugarcane

## Abstract

Manganese toxicity has limited sugarcane (*Saccharum* spp. hybrid.) growth and production in acidic soils in south China. The rhizosphere plays an irreplaceable role in plant adaptation to soil abiotic stress, but the responses of the sugarcane rhizosphere to manganese toxicity are still unknown. We designed pot experiments in Mn-rich acidic soil, collected the sugarcane rhizosphere and bulk soil samples, and then investigated the changes in Mn-related soil parameters and microbiome. The results indicated that the water-soluble and exchangeable manganese concentrations in the sugarcane rhizosphere were significantly lower than that in the bulk soil, which was not associated with soil pH changes. In contrast, the number of bacteria and the activity of peroxidase, sucrase, urease, and laccase in the rhizosphere were significantly higher. The 16S rDNA sequencing results showed that the bacterial diversity and quantity along with the abundance of *Proteobacteria* in the rhizosphere were significantly higher than in the bulk soil, while the abundance of *Acidobacteria* was lower than in the bulk soil. The soil laccase activity and the number of bacteria decreased significantly with the increase in the manganese toxicity stress. Finally, the relative abundance of proteins associated with manganese transportation and oxidation was significantly higher in the rhizosphere soil. In summary, the Mn-induced response of the rhizosphere is an important mechanism in sugarcane adaptation to manganese toxicity in acidic soil.

## 1. Introduction

Manganese (Mn) is an essential micronutrient for plant growth and development, including photosynthesis, respiration, protein synthesis, and hormone activation [1]. But Mn can be toxic to plants when present in excess [2]. Excessive Mn can interfere with the absorption, translocation, and utilization of other essential elements, inhibit enzyme activity, induce oxidative damage, and ultimately inhibit photosynthesis and plant growth [3]. In fact, Mn toxicity is considered to be the second largest plant-growth-limiting factor after aluminum toxicity in acidic soils [4]. Plants generally have two mechanisms to cope with Mn toxicity: cellular tolerance and transport and uptake regulation [5,6]. Mn-tolerant plants can upregulate transporters like *MTP8.1* and *MTP11* to compartmentalize Mn into subcellular compartments to withstand the toxic effects of excessive Mn stress [7,8]. For instance, previous research showed that Mn sequestration into the cell wall was involved in Mn tolerance in sugarcane (*Saccharum officinarum* L.) [9]. On the other hand, preventing excessive Mn from entering the plant by regulating uptake is the first step in dealing with Mn toxicity. Soil Mn is present in multiple valence states, ranging from Mn^2+^ to Mn^7+^ [3]. Mn^2+^, in the most commonly occurring water-soluble and exchangeable form, has the highest availability for plant acquisition [10]. Thus, reducing the bioavailability of Mn in soil, especially rhizosphere soil, may also contribute to overcoming Mn toxicity for plants grown in acidic soils.

The rhizosphere is the microhabitat where microorganisms communicate with plants through chemical messages and develop synergistic actions [11]. Healthy rhizosphere microbial communities promote plant productivity and the capacity to respond to stressful environments [12,13]. Many studies have reported significant differences between the plant rhizosphere soil and the surrounding bulk soil under different abiotic stresses such as heat, drought, and certain heavy metals [14,15,16,17]. Rhizosphere microbes can transform heavy metals into less toxic forms and alter their availability. For example, microbial oxidation and adsorption by Mn-oxidizing bacterial strains offer novel approaches to removing Mn pollution [18]. Mn bio-oxidation follows direct methods like the effects of related enzymes and/or indirect methods like the improvement in pH and redox conditions [19]. However, there is very little research on their potential effect on plants growing in acidic and Mn-rich soil, and there are no reports on the effects of the rhizosphere on sugarcane’s responses to Mn toxicity. Sugarcane is an important crop that can be used for biofuel production and has a high economic value worldwide [20]. Recently, sugarcane grown in acid soils in south China has been severely affected by Mn toxicity [4]. Our research has demonstrated that sugarcane grown in soil pots typically exhibits a higher Mn tolerance than that grown in hydroponic experiments. Therefore, studying the rhizosphere’s mechanisms that contribute to alleviating Mn toxicity stress in sugarcane is of great practical value.

This study applied additional Mn or calcium carbonate to soils with high manganese background concentrations to evaluate the toxicity effects of different Mn concentrations. Excess Mn^2+^ in soil (mainly including water soluble and exchangeable Mn) causes toxicity to plants [4], while decreased Mn^2+^ levels alleviate the potential toxicity. And research has reported that applying calcium carbonate will significantly decrease soil Mn toxicity [21]. The rhizosphere and bulk soil were collected from sugarcane plants, and their physical and chemical properties were compared, as well as their microbial community composition and structure. The results revealed the role of the sugarcane rhizosphere under Mn stress and can provide a research foundation for the further application of effective strategies to minimize Mn toxicity in acidic soils.

## 2. Materials and Methods

### 2.1. Experiment Design

**Figure 1:**
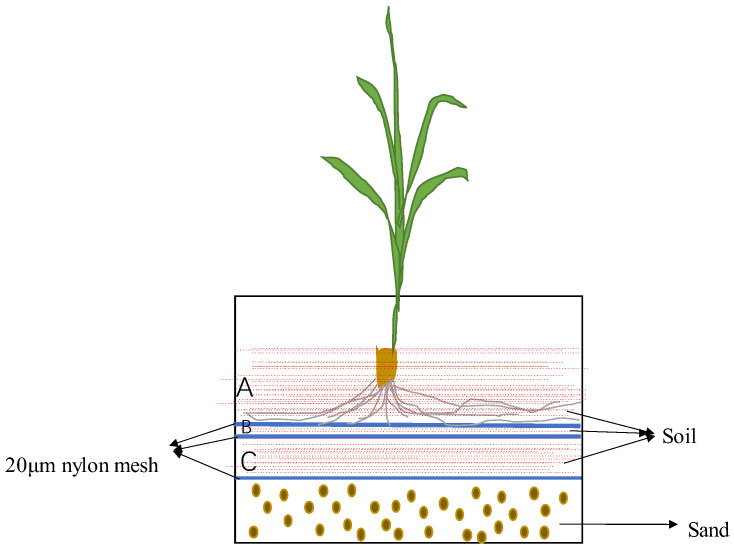
Pad planting sketch map in this experiment. Layer A was planting layer, layer B was considered as rhizosphere soil, layer C was considered as bulk soil.

The soil in this experiment was collected from sugarcane fields where Mn-induced chlorosis had occurred continuously year after year in Chongzuo, Guangxi Province, China. The initial soil physicochemical parameters were as follows: pH 4.5, soil organic matter 18.7 g/kg, available N 60.6 mg/kg, available P 12.58 mg/kg, and available K 127.66 mg/kg. Sugarcane ZHONGZHE 1 cultivar seedlings were sown, ensuring that one seedling grew. Basal doses of N at 120 mg kg^−1^ soil, P_2_O_5_ at 60 mg kg^−1^ soil, and K_2_O at 33 mg kg^−1^ soil were applied. Considering that liming and addition of Mn salt and calcium carbonate [21] can change soil Mn availability and toxicity, different levels of Mn stress were set up to investigate the impact of Mn stress on the rhizosphere microbial community of sugarcane, and four treatments were set up in the experiment: ① the original Mn concentration in the sampled soil (Mn0), ② the original Mn concentration in the soil +0.5 g Mn/kg soil (Mn0.5), ③ the original Mn concentration in the soil +1.0 g Mn/kg soil (Mn1), and ④ the original Mn concentration in the soil +10 g CaCO_3_/kg soil (Ca). Each treatment was carried out with three biological replicates. To collect the rhizosphere and bulk soil more accurately, pad planting was applied in this experiment (Figure 1). The plants grew in a controlled-environment growth chamber with a 14 h (30 °C)/10 h (25 °C) day/night cycle, and the relative humidity was kept at 75–85%. The daytime light intensity was 400 μmol m^−2^ S^−1^. Every 5 days, the plants were watered to support sugarcane growth. After growing for 70 days (at which point the roots completely covered the bottom of the planting layer), the experiment was completed.

### 2.2. Sample Collection

After excavating layer A of each treatment and carefully removing the nylon mesh, the soil in layer B was thoroughly mixed. Subsequently, sterilized bags were used to collect 2.0–3.0 g of soil samples from the rhizosphere soil of each treatment. Additionally, 2.0–3.0 g of soil samples were collected from the bulk soil of each treatment after evenly mixing all the soil in layer C. These soil samples were stored in low-temperature refrigerated boxes and transported to the laboratory for further analysis. The samples were stored in a −80 °C ultra-low-temperature refrigerator to prepare them for microbial sequencing. Moreover, some samples were temporarily stored in a 4 °C low-temperature freezer to facilitate various analyses that would be conducted shortly after sampling.

### 2.3. Determination of Soil pH, Water-Soluble Mn, Exchangeable Mn, and Enzyme Activities

The soil pH was determined by the potentiometric method (soil/purified water = 2.5/1) with a pH meter. Soil water-soluble Mn (WS–Mn) was extracted with purified water, and soil exchangeable Mn (EX–Mn) was extracted with 1 mol L^−1^ NH_4_OAc (pH 7.0) for 30 min, then measured by AAS [4]. Activities of soil peroxidase (S–POD), sucrase (S–SC), urease (S–UE), and laccase (S–L) were determined with a spectrophotometer using assay kits (Solarbio, Beijing, China).

### 2.4. DNA Extraction and Sequencing of Partial 16S rRNA Genes

The soil DNA from all samples was extracted from 0.50 g of soil using the E.Z.N.A™ Mag–Bind Soil DNA Kit (OMEGA, M5635–02,Shanghai, China). After precise quantification of genomic DNA using the Qubit3.0 DNA Kit, the V3–V4 regions of the bacterial 16S rRNA gene were amplified by using the primers 338F (5′–ACTCCTACGGGAGGCAGCAG–3′) and 806R (5′–GGACTACHVGGGTWTCTAAT–3′). The following PCR program was used as follows: initial denaturation at 94 °C for 3 min, 5 cycles of denaturation at 94 °C for 30 s, 45 °C 20 s, 65 °C 30 s; 20 cycles of 94 °C for 20 s, 55 °C for 20 s, and 72 °C 30 s, then extension at 72 °C for 5 min with a thermocycler PCR system (ETC 811, Dongsheng, Beijing, China). The PCR products were sent to Sangon Biotech (Shanghai, China). The libraries were sequenced using an Illumina HiSeq platform (HiSeq SBS Kit V2, Illumina, San Diego, CA, America).

## 3. Results

### 3.1. pH Values in the Rhizosphere and Bulk Soil

**Figure 2:**
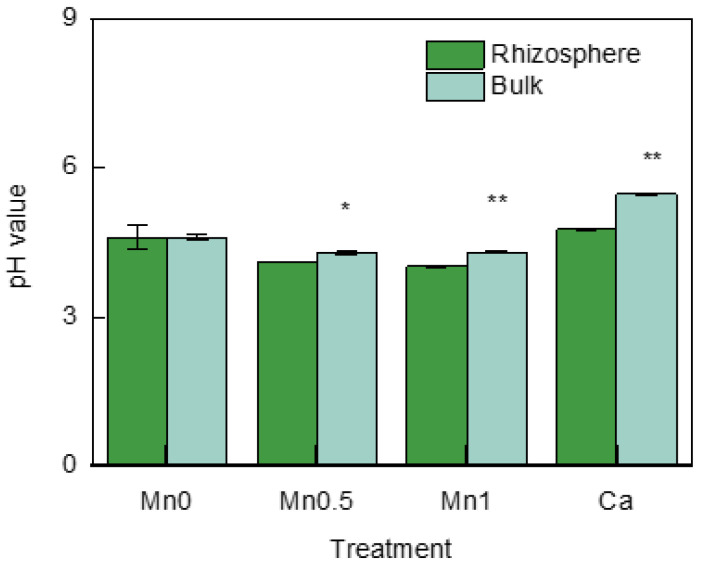
pH values in the rhizosphere and bulk soil in different treatments; * means significant at *p* < 0.05; ** significant at *p* < 0.01.

Based on the results (Figure 2), with the exception of the Mn0 treatment, the other three treatments showed a significant increase in the pH of the bulk soil compared with the rhizosphere soil. The pH values of the rhizosphere soil and bulk soil in the Mn0 treatment were 4.59 and 4.60, respectively. The pH of the rhizosphere soil in the Mn0.5 treatment was 4.09, while that of the bulk soil was 4.30, decreased by 0.21 units. The pH of the rhizosphere soil in the Mn1 treatment was 4.01, decreased by 0.28 compared with 4.29 in the bulk soil. The pH of the rhizosphere soil in the Ca treatment was 4.76, decreased by 0.72 units compared with the bulk soil, which had a pH of 5.47.

### 3.2. WS–Mn and EX–Mn Concentration in the Rhizosphere and Bulk Soil

**Figure 3:**
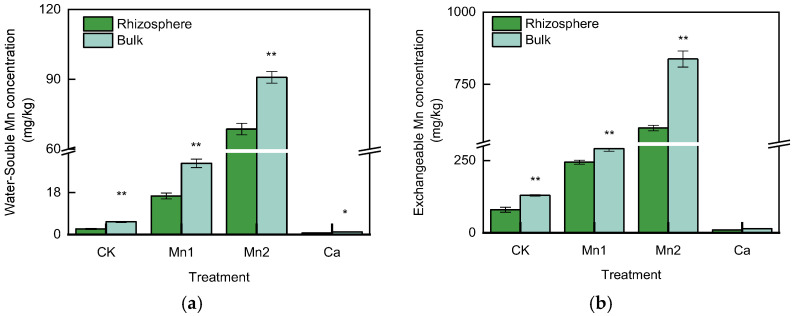
Mn concentration in the rhizosphere and bulk soil in different treatments. (**a**) Water-soluble Mn (WS–Mn); (**b**) exchangeable Mn (EX–Mn); * means significant at *p* < 0.05; ** significant at *p* < 0.01.

As shown in Figure 3a, the concentration of water-soluble Mn (WS–Mn) in the rhizosphere soil was 2.40 mg/kg, which was 55.9% lower than that of the bulk soil at 5.43 mg/kg in the Mn0 treatment. In the Mn0.5 treatment, the WS–Mn concentration in the rhizosphere soil was 16.54 mg/kg and 30.58 mg/kg in the bulk soil, decreased by 46.0%. The WS–Mn concentration in the rhizosphere soil in Mn1 was 68.615 mg/kg, decreased by 24.5% compared with the bulk soil, at 90.88 mg/kg. In the Ca treatment, the WS–Mn concentration in the rhizosphere soil was 0.63 mg/kg, 41.2% lower than that of the bulk soil at 1.07 mg/kg. The differences in WS–Mn concentration between the rhizosphere and bulk soil in all treatments were significant.

As shown in Figure 3b, the exchangeable Mn (EX–Mn) concentration in the rhizosphere soil of the Mn0 treatment was 79.75 mg/kg, 38.4% lower than that of the bulk soil, which was 129.5 mg/kg. In the Mn0.5 treatment, the EX–Mn in rhizosphere soil was 244.06 mg/kg, decreased by 16.1% compared with the bulk soil at 290.96 mg/kg. The EX–Mn in the rhizosphere soil of the Mn1 treatment was 599.12 mg/kg; in the bulk soil, it was 837.78 mg/kg, indicating that it decreased by 28.5%. In the Ca treatment, the EX–Mn concentration in the rhizosphere soil was 9.60 mg/kg, 32.0% lower than that of the bulk soil at 14.12 mg/kg. Mn0, Mn0.5, and Mn1 treatment showed significant differences in EX–Mn between the rhizosphere and bulk soil. In all treatments, WS–Mn and EX–Mn concentrations in the rhizosphere soil were significantly lower than in the bulk soil.

It can be concluded that the sugarcane rhizosphere decreased soil WS–Mn and EX–Mn concentration.

### 3.3. Soil Enzyme Activities in the Rhizosphere and Bulk Soil

**Figure 4:**
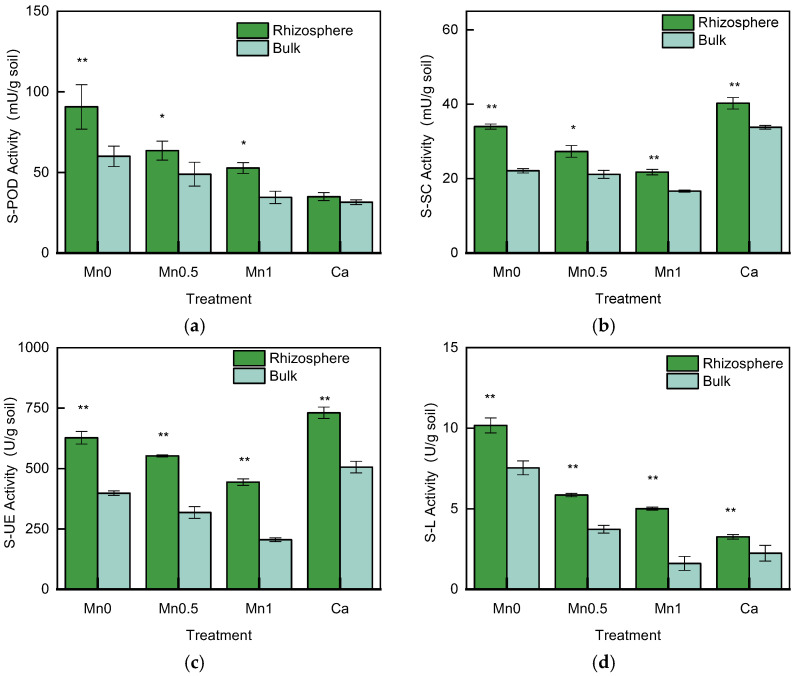
Soil enzyme activities in the rhizosphere and bulk soil in different treatments; (**a**) peroxidase (S–POD), (**b**) sucrase (S–SC), (**c**) urease (S–UE), and (**d**) laccase (S–L). * means significant at *p* < 0.05; ** significant at *p* < 0.01.

The soil peroxidase (S–POD), sucrase (S–SC), urease (S–UE), and laccase (S–L) activity showed highly significant differences in the rhizosphere and bulk soil (Figure 4). The S–POD activity in the rhizosphere of the Mn0 treatment increased by 51.2% compared with the bulk soil. In the Mn0.5 treatment and Mn1 treatment, it increased by 29.9% and 52.9%, respectively. The S–SC activity in the Mn0 treatment was increased by 53.7% in the rhizosphere soil compared with the bulk soil, by 29.3% in Mn0.5, by 30.6% in Mn1, and by 19.1% in the Ca treatment. The S–UE activity increased by 57.6% in the rhizosphere soil of the Mn0 treatment compared with the bulk soil, while it was increased by 73.7% in Mn0.5, by 116.1% in Mn1, and by 44.5% in the Ca treatment. The S–L activity in the rhizosphere soil of the Mn0 treatment was increased by 34.9% compared with the bulk soil, by 57.0% in Mn0.5, by 211.1% in Mn1, and by 45.4% in the Ca treatment.

### 3.4. Comparison of the Number of Cultivable Bacteria in the Rhizosphere and Bulk Soil

**Figure 5:**
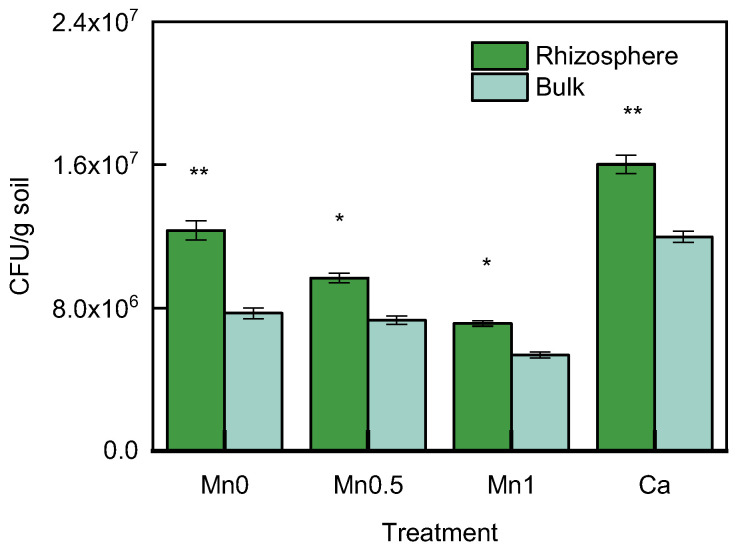
Colony-forming unit (CFU) in the rhizosphere and bulk soil in different treatments; * means significant at *p* < 0.05; ** significant at *p* < 0.01.

The cultivable colony-forming unit (CFU) counts of bacteria in the rhizosphere soil and bulk soil were evaluated for each treatment (Figure 5). The Mn0 treatment had a CFU value of 1.23 × 10^7^, which was increased by 60.1% compared with the bulk soil CFU at 7.70 × 10^6^. The Mn0.5 treatment rhizosphere soil had a CFU value of 9.68 × 10^6^, increased by 32.2% compared with the bulk soil CFU at 7.31 × 10^5^. The Mn1 treatment rhizosphere soil had a CFU value of 7.13 × 10^6^, increased by 32.7% compared with the bulk soil CFU of 5.37 × 10^6^. The Ca treatment rhizosphere soil had a CFU value of 1.60 × 10^7^, increased by 33.7% compared with the bulk CFU of 1.20 × 10^6^. As seen from the above results, the number of bacteria in the rhizosphere soil was significantly higher than in the bulk soil in all treatments.

**Figure 6:**
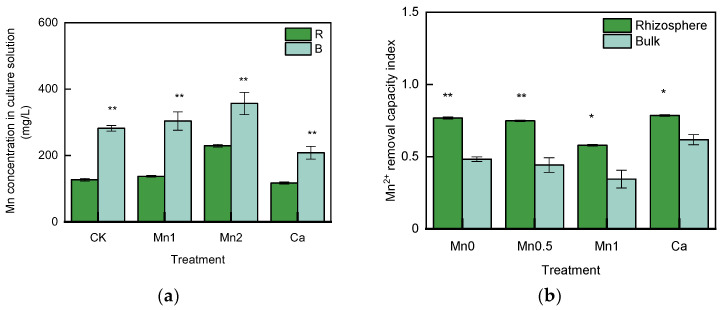
Mn^2+^ removed by bacteria in the rhizosphere and bulk soil in different treatments; (**a**) residual Mn concentration in culture solution; (**b**) Mn^2+^ removal ability index. * means significant at *p* < 0.05; ** significant at *p* <0.01.

As shown in Figure 6a, the residual Mn concentration in the bulk soil in all treatments was significantly higher than that in the rhizosphere soil. Compared with the bulk soil samples, the residual Mn concentration in the rhizosphere soil decreased by 55.0% in the Mn0 treatment, 54.8% in the Mn0.5 treatment, 35.7% in the Mn1 treatment, and 43.7% in the Ca treatment. And as shown in Figure 6b, the Mn removal ability in rhizosphere soil was significantly higher than that of the bulk soil in all treatments.

### 3.5. 16S rDNA Sequencing in the Rhizosphere and Bulk Soil

In Mn0, Mn0.5, and Mn1 treatment, the Shannon diversity index of the rhizosphere soil showed a significant increase when compared with that of the bulk soil (Table 1). Conversely, the Simpson index of the rhizosphere soil was lower than that of the bulk soil. No differences were observed between the rhizosphere and bulk soil in the Ca treatment. The Chao index of the rhizosphere soil was significantly higher than that of the bulk soil in all treatments. Finally, by comparing the Shannoneven index (which represents the evenness of community distribution), it was found that rhizosphere soil had significantly higher values than the bulk soil in Mn0 and Mn0.5. Although the Shannoneven index of the rhizosphere soil was also higher than that of the bulk soil in Mn1 and Ca treatment, the differences were not significant. The results indicated that the number, diversity, and evenness of rhizosphere soil bacteria increased in the sugarcane rhizosphere soil compared with the bulk soil under Mn toxicity.

**Figure 7:**
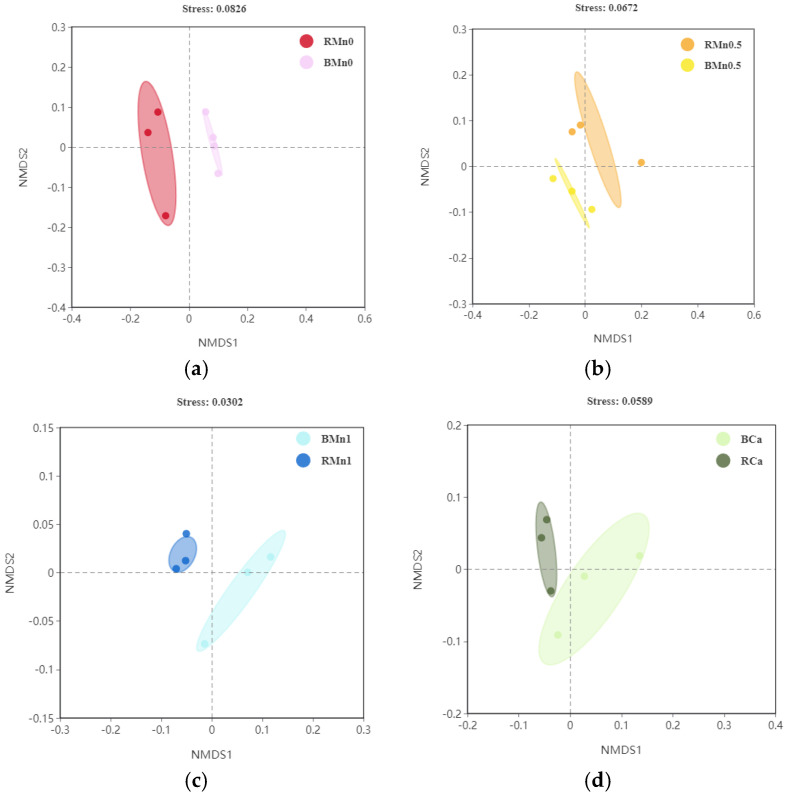
Non-metric multidimensional scaling (NMDS) analysis in the rhizosphere and bulk soil in different treatments at phylum level; (**a**) Mn0 treatment; (**b**) Mn0.5 treatment; (**c**) Mn1 treatment; (**d**) Ca treatment.

The analysis of NMDS results (Figure 7) revealed that the bacterial communities in the rhizosphere and bulk soil in all treatments were separated along the NMDS1 axis. There was a certain distance between the rhizosphere and bulk soil samples in Mn0, Mn0.5, and Mn1 treatment, indicating a certain difference in the structural composition of bacterial communities in the rhizosphere and bulk soil in these treatments.

**Figure 8:**
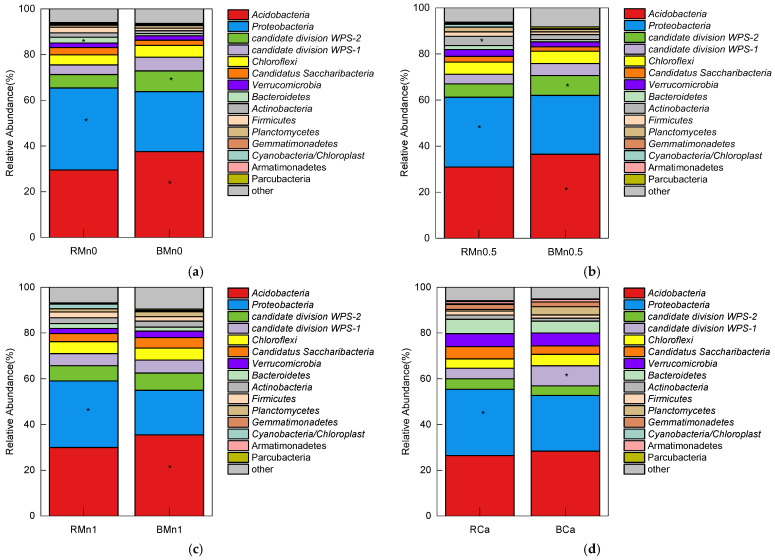
Relative abundance of the dominant phyla in the rhizosphere and bulk soil in different treatments. (**a**) Mn0 treatment; (**b**) Mn0.5 treatment; (**c**) Mn1 treatment; (**d**) Ca treatment. * means significant at *p* < 0.05.

There was no difference in the composition between the rhizosphere and bulk soil at the phylum level, but the relative abundances of species were different (Figure 8). In the Mn0, Mn0.5, and Mn1 treatment, the relative abundance of *Acidobacteria* in the rhizosphere soil was 29.51%, 30.89%, and 29.94%, respectively, significantly lower than that in the bulk soil (37.58%, 36.5%, and 35.47%). In the Mn0, Mn0.5, Mn1, and Ca treatment, the relative abundance of *Proteobacteria* in the rhizosphere soil was 35.9%, 30.32%, 29.09%, and 28.68%, respectively, significantly higher than that in the bulk soil (26.21%, 25.55%, 19.51%, and 24.31%). The *candidate division WPS–2* abundance in the rhizosphere soil treated with Mn0 and Mn0.5 was 5.8% and 5.8%, respectively, lower compared with 9.1% and 8.6% in the bulk soil.

**Figure 9:**
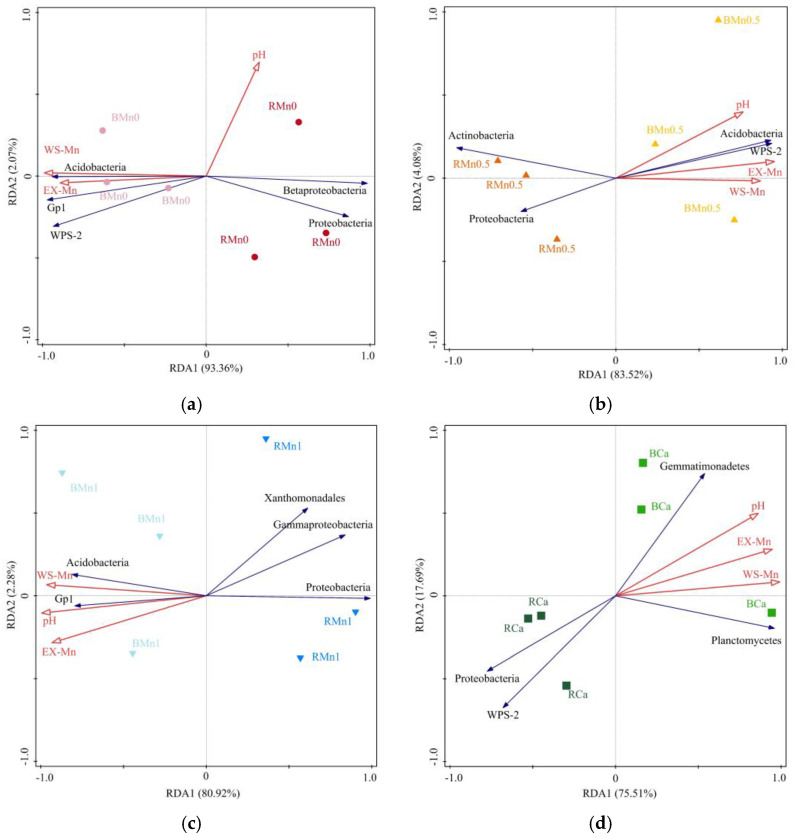
Redundancy analysis (RDA) of soil bacterial community composition and soil WS–Mn, EX–Mn, and pH; (**a**) Mn0 treatment; (**b**) Mn0.5 treatment; (**c**) Mn1 treatment; (**d**) Ca treatment.

The influences of soil physicochemical properties on the bacterial communities of each treatment were analyzed (Figure 9). WS–Mn and EX–Mn in the soil had a positive correlation with the abundance of *Acidobacteria*, *WPS–2*, and *Gp1* in Mn0, Mn0.5, and Mn1 treatment, while they had a negative correlation with *Proteobacteria* in all treatments and with *Betaprotebacteria* in Mn0 treatment and *Actinobacteria* in Mn0.5 treatment. There was also a negative correlation between *Gammaproteobacteria* and *Xanthomonadales* in Mn1 treatment. The pH value was positively correlated with the abundance of *Acidobacteria*, *WPS–2*, and *Gp1* in Mn0.5 and Mn1 treatments while it was negatively correlated with the abundance of *Proteobacteria* in Mn0.5, Mn1, and Ca treatment.

**Figure 10:**
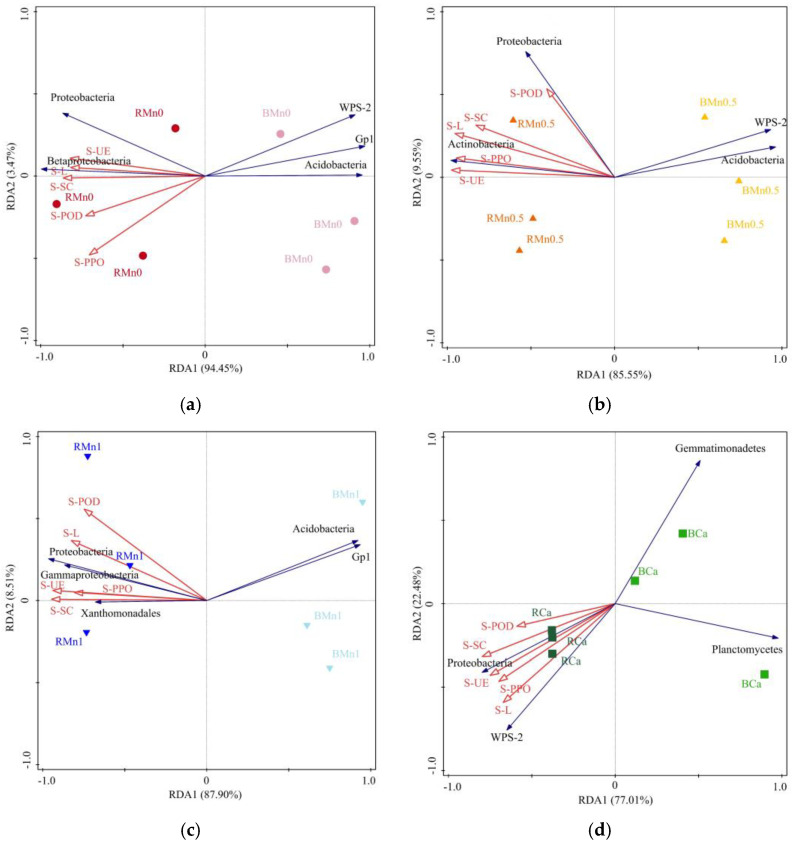
Redundancy analysis (RDA) of soil bacterial community composition and S–UE, S–SC, S–POD, and S–L enzyme activities; (**a**) Mn0 treatment; (**b**) Mn0.5 treatment; (**c**) Mn1 treatment; (**d**) Ca treatment.

Furthermore, Figure 10 shows S–UE, S–SC, S–POD, and S–L had a positive correlation with the abundance of *Proteobacteria* in each treatment, while they had a negative correlation with the abundance of *Acidobacteria* in Mn0, Mn0.5, and Mn1 treatment and *Acidobacteria Gp1* in Mn0 and Mn1 treatment. In addition, the activity of each enzyme was positively correlated with the abundance of Betaprotebacteria in Mn0 treatment, *Actinobacteria* in Mn0.5 treatment, *Gammaprotebacteria* in Mn1 treatment, and *WPS–2* in Ca treatment. There was a negative correlation between the abundance of *WPS–2* and soil enzyme activities in Mn0 and Mn0.5 treatment, as well as the abundance of *Xanthomonas* in Mn1 treatment.

**Figure 11:**
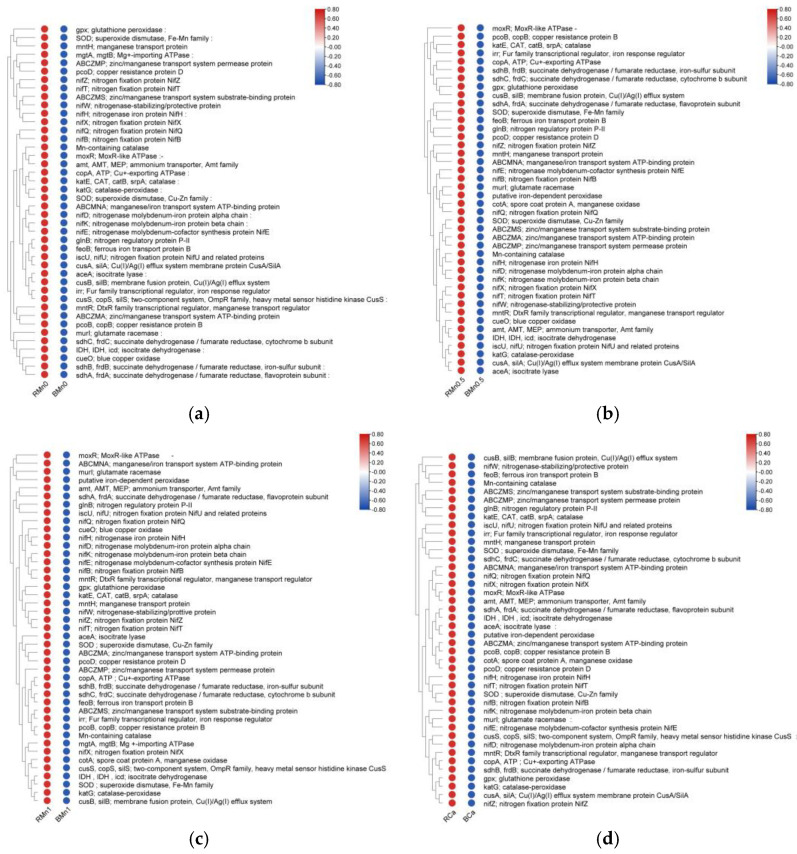
Heatmap of KEGG orthologs’ relative expression in the rhizosphere and bulk soil in different treatments; (**a**) Mn0 treatment; (**b**) Mn0.5 treatment; (**c**) Mn1 treatment; (**d**) Ca treatment.

A total of 6767 KEGG orthologs (KOs) were matched to the annotations. The relative expressions of proteins related to Mn transportation, such as *mntH* and *mntR*, copper oxidation such as *copA*, *cusA*, and *cusS*, Mn oxidation-related proteins, such as *Mn-containing catalase* and *SOD*, and *nifZ* and *nifX* related to nitrogen metabolism in the rhizosphere soil were significantly higher than those in the bulk soil across all treatments (Figure 11). These proteins were reported as being involved in Mn biooxidation and regulation [19]. And it can be concluded that the sugarcane rhizosphere activated soil bacteria-related functions related to adaptation to excess Mn stress.

## 4. Discussion

Soil Mn chemical forms mainly include Mn^2+^, Mn^3+^, and Mn^4+^. Only Mn^2+^ in the soil solution can be absorbed by plants, while Mn^3+^ and Mn^4+^ are highly insoluble and will form precipitates in the soil [22]. Therefore, reducing the Mn^2+^ concentration in soil will be beneficial for alleviating Mn toxicity to plants. Mn^2+^ mainly exists in the soil as WS–Mn and EX–Mn. The WS–Mn and EX–Mn concentrations were analyzed in the rhizosphere and bulk soil. In each treatment, the WS–Mn and EX–Mn concentrations in the rhizosphere soil were significantly lower than in the bulk soil. A similar result has been reported in white lupin, in which the rhizosphere soil concentrations of 0.1 M CaCl_2_-extractable Mn, Zn, and Cu were lower than in the bulk soil [23]. In acidic lateritic red soil, the pH strongly influenced WS–Mn and EX–Mn concentrations, showing a significant negative correlation [4]. The pH values were measured to verify whether the differences in WS–Mn and EX–Mn between the rhizosphere and bulk soil in all treatments were caused by changes in pH. It was found that there was no difference in pH values between the rhizosphere and bulk soil in Mn0 treatment, while the pH values of the rhizosphere in Mn0.5, Mn1, and Ca treatment were lower than those in the bulk soil. Therefore, it could be concluded that there existed a mechanism in the rhizosphere to alleviate Mn toxicity, which was not associated with pH changes.

In this experiment, the lower pH value in the sugarcane rhizosphere soil compared with the bulk soil may be related to the secretion of organic acids and other substances by plant roots. During the growth process of oats, the root system secretes a wide range of metabolites such as sugars, amino acids, organic acids, etc. [14]. And barley root-exudated organic acid was considered as a mechanism associated with Mn alleviation [24]. Our previous studies have found that sugarcane secreted vitamin B group substances such as niacin and nicotinamide under Mn toxicity stress, which may be related to the low pH value of rhizosphere soil in this experiment.

S–POD, S–UE, S–SC, and S–L activities in rhizosphere soil were significantly higher when compared with the bulk soil. Soil enzyme activities are closely related to soil nutrient conversion and microbial metabolism, and plant roots will adjust according to environmental changes during their growth, thus affecting soil enzyme activity [25]. S–POD is related to the synthesis and decomposition of soil organic matter, and S–POD activity has an important impact on the soil carbon cycle. S–UE and S–SC are positively correlated with the quantity and community composition of soil microorganisms. S–SC can catalyze the hydrolysis of sucrose to glucose, which is beneficial for soil organisms to obtain carbon sources. At the same time, S–UE can hydrolyze urea; thus, it is involved in soil nitrogen metabolism [26]. In addition to the microbial degradation of lignin, S–L is an important Mn peroxidase in soil, which is involved in the oxidation process of Mn [27]. Enzyme activities in the rhizosphere soil in all treatments were significantly higher than those in the bulk soil, indicating that the effects of microbial activity, nutrient transformation, and Mn metabolism in rhizosphere soil were stronger than those in the bulk soil.

The results indicated that the number of cultivable bacteria (CFU) in all treatments of the rhizosphere soil was significantly higher than in the bulk soil. Moreover, the results of the 16S rDNA sequencing further confirmed that differences existed in microbial diversity between the rhizosphere and bulk soil samples. Alpha diversity analysis showed that the number and diversity of bacteria in rhizosphere soil were significantly higher than in the bulk soil, and the different soil samples were clearly separated, indicating a significant difference in the composition of the rhizosphere and bulk soil samples. The similar result has been found in rice rhizosphere when under mixed heavy metal contamination, which displayed higher bacterial diversity indices than the bulk soil [28], and *Robinia pseudoacacia* L. rhizosphere bacterial species richness was higher than that of the bulk soil under heavy metal contamination [29]. This might be due to the impact of the sugarcane rhizosphere, which increased the number of soil bacteria. Based on previous results, it can be concluded that the rhizosphere environment of sugarcane promoted an increase in the number of microorganisms in the soil under various Mn concentrations, promoted microbial removal of Mn, and thus reduced the concentration of WS–Mn and EX–Mn in the rhizosphere soil.

In terms of bacterial species abundance, there were significant differences between the rhizosphere and bulk soil across the different treatments. The abundance of *Proteobacteria* in rhizosphere soil was significantly higher than in the bulk soil, while *Acidobacteria* were significantly lower in abundance. *Proteobacteria* play a promoting role in plant growth and can promote plant growth under adverse conditions [30], showed adaptation to high concentrations of heavy metals, and can immobilize heavy metals [31]. Cultivable members of *Acidobacteria* are considered oligotrophic bacteria [32,33]. Notably, the abundance of *Acidobacteria* in the rhizosphere of Chinese cabbage in chromium-contaminated soil was significantly lower than that in the bulk soil [32]. When under stress, the abundance of *Acidobacteria* is usually quite high [34,35,36]. Furthermore, we analyzed the effects of soil WS–Mn, EX–Mn, and enzyme activities on soil microbial species composition. The WS–Mn and EX–Mn were significantly negatively correlated with the abundance of *Proteobacteria*, while they were significantly positively correlated with the abundance of *Acidobacteria*. The activity of each enzyme was positively correlated with the abundance of *Proteobacteria*, while it was significantly negatively correlated with the abundance of *Acidobacteria*. Based on the change in soil Mn concentration, the change in *Proteobacteria* and *Acidobacteria* abundance may play important roles in the reduction of toxic forms of manganese in the rhizosphere of sugarcane.

Proteins related to Mn transportation [37], Mn oxidation [38,39,40,41], and redox reactions [42,43] were significantly upregulated in relative expression in the rhizosphere soil [44,45]. This may indicate there was a significantly increased in rhizosphere bacterial relative functions. Moreover, the relative abundance of proteins related to the conversion of nitrate nitrogen to ammonium nitrogen also significantly increased in the rhizosphere soil, and previous studies have found that ammonium nitrogen is beneficial for the alleviation of Mn toxicity in sugarcane [46]. In conjunction with the WS–Mn and EX–Mn concentration in the rhizosphere soil and bacterial Mn removal ability, this indicated that the activity of these proteins is beneficial for microorganisms to adapt to Mn stress and reduce the availability of Mn^2+^. Similar results have been reported in other studies: resistance genes (*piuA, copA, aoxB, arsC, CzrB, ZIP*, etc.)contributed to the survival of rhizosphere microbes by promoting the adaptation to heavy metal stress [47].

## 5. Conclusions

This study found that the Mn-induced response of the rhizosphere is an important mechanism in sugarcane adaptation to manganese toxicity in acidic soil. Compared with the bulk soil, the growth of bacteria, soil enzyme activities, and bacterial diversity increased significantly in the rhizosphere. Significant increases in abundance of *Proteobacteria* and relative abundance of proteins related to Mn transportation and oxidation were also observed in the rhizosphere. These differences may be attributed to the secretion of metabolites in the sugarcane rhizosphere, which regulated the soil microenvironment to reduce excess Mn toxicity.

## Figures and Tables

**Table 1:** Alpha diversity index in the rhizosphere and bulk soil in different treatments.

Treatment	Region	Shannon	Simpson	Chao	Shannoneven
Mn0	Rhizosphere	3.51 ± 0.06 **	0.049 ± 0.002	100.67 ± 5.81 *	0.76 ± 0.003 *
	Bulk	3.16 ± 0.03	0.070 ± 0.002 **	77.33 ± 0.33	0.73 ± 0.007
Mn0.5	Rhizosphere	3.52 ± 0.05 *	0.049 ± 0.002	96.33 ± 3.48 *	0.78 ± 0.011 *
	Bulk	3.23 ± 0.05	0.067 ± 0.003 **	83.00 ± 2.89	0.73 ± 0.005
Mn1	Rhizosphere	3.42 ± 0.02 *	0.051 ± 0.001	86.67 ± 3.28 *	0.77 ± 0.009
	Bulk	3.25 ± 0.10	0.066 ± 0.007 *	76.00 ± 4.00	0.75 ± 0.013
Ca	Rhizosphere	3.50 ± 0.00	0.047 ± 0.002	95.00 ± 1.73 *	0.76 ± 0.002
	Bulk	3.50 ± 0.03	0.046 ± 0.002	85.67 ± 1.86	0.76 ± 0.020

Values represent the mean ± S.E. (n = 3); * means significant at *p* = 0.05; ** significant at *p* = 0.01.

## Data Availability

The data and trial protocol can be requested from the corresponding authors.
